# Evaluation of Poxvirus-Specific Antibody Response in Monkey Poxvirus-Negative and -Positive Cohorts

**DOI:** 10.3390/vaccines13080795

**Published:** 2025-07-27

**Authors:** Nannan Jia, Lin Ai, Yunping Ma, Chen Hua, Qi Shen, Chen Wang, Teng Li, Yingdan Wang, Yunyi Li, Yin Yang, Chi Zhou, Min Chen, Huanyu Wu, Xin Chen, Lu Lu, Yanqiu Zhou, Jinghe Huang, Fan Wu

**Affiliations:** 1 Shanghai Immune Therapy Institute, Ren Ji Hospital, Shanghai Jiao Tong University School of Medicine, Shanghai 200127, China; jnn0106@sjtu.edu.cn (N.J.); wchen94@sjtu.edu.cn (C.W.); liteng2024@sjtu.edu.cn (T.L.); no.1yy@126.com (Y.Y.); zhouchi@renji.com (C.Z.); 2 Institute of Microbiology Laboratory, Shanghai Municipal Center for Disease Control and Prevention, Shanghai 201100, China; ailin@scdc.sh.cn (L.A.); shenqi_2@scdc.sh.cn (Q.S.); liyunyi@scdc.sh.cn (Y.L.); chenmin@scdc.sh.cn (M.C.); wuhuanyu@scdc.sh.cn (H.W.); chenxin@scdc.sh.cn (X.C.); 3 Key Laboratory of Medical Molecular Virology (MOE/NHC/CAMS) and Shanghai Institute of Infectious Disease and Biosecurity, School of Basic Medical Sciences, Fudan University, Shanghai 200032, China; ypma23@m.fudan.edu.cn (Y.M.); hua_chen@fudan.edu.cn (C.H.); yingdanwang@fudan.edu.cn (Y.W.); lul@fudan.edu.cn (L.L.)

**Keywords:** monkey poxvirus, neutralizing antibodies, MSM, ADCC

## Abstract

Objectives: Understanding the antibody response in monkeypox virus (MPXV)-infected and uninfected individuals is essential for developing next-generation MPXV vaccines. This study aimed to characterize neutralizing antibody (NAb) and antibody-dependent cellular cytotoxicity (ADCC) responses in both groups, providing insights into immune protection and vaccine design. Methods: A recombinant vaccinia Tian Tan (VTT) virus was utilized to develop high-throughput luciferase-reporter-based neutralization and ADCC assays. These assays were applied to evaluate the presence and levels of poxvirus-specific antibodies in MPXV-infected and uninfected individuals, including those vaccinated with vaccinia-based vaccines. Results: Poxvirus-specific NAbs were detected in MPXV-negative individuals with prior vaccinia vaccination. However, MSM individuals exhibited significantly lower pre-existing NAb levels than non-MSM individuals, potentially contributing to their higher susceptibility to MPXV infection. In individuals with mild MPXV infection, robust NAb and ADCC responses were observed, regardless of vaccination status. Additionally, HIV-positive individuals demonstrated comparable antibody responses following MPXV infection. Conclusions: These findings highlight the potential role of pre-existing NAbs in MPXV susceptibility and the strong immune response elicited by mild MPXV infection. Further research is needed to determine whether MPXV-specific antibodies mitigate disease progression, which could inform the development of effective MPXV vaccines.

## 1. Introduction

Orthopoxviruses, including variola, cowpox, horsepox, camelpox, and monkeypox viruses, are a group of large, enveloped DNA viruses that infect vertebrates, including humans [1]. The variola virus, responsible for smallpox, was one of the most devastating diseases in human history, resulting in millions of fatalities. Smallpox was successfully eradicated by a global vaccination campaign with the vaccinia virus in 1980 [2]. Following this achievement, the smallpox vaccination program was discontinued. Since 2022, the global mpox outbreak has emerged, causing significant public health impacts worldwide [3]. Infected individuals have predominantly presented with mild symptoms, such as rash, fever, headache, and muscle pain [4]. Severe and fatal cases were usually observed in patients with immunodeficiency such as HIV-infected individuals. Over 100 countries have reported MPXV cases between January 2022 and December 2024, with more than 100,000 laboratory-confirmed cases and over 200 deaths. This outbreak has underscored the urgency of addressing this public health challenge [5]. Consequently, the urgent need to develop effective vaccines and therapeutic interventions against MPXV and related poxviruses has gained significant attention.

Vaccinia-based vaccines, including vaccinia Tian Tan (VTT) and modified vaccinia Ankara (MVA), have proven effective against the smallpox virus [3]. These vaccines induce robust and long-lasting antibody and T-cell responses following immunization [6]. For smallpox, antibodies were considered the primary correlate of protection for vaccinia vaccines. Neutralizing antibody (NAb) titers serve as the gold standard for assessing smallpox vaccine efficacy [7]. Serum NAb titers exceeding 1:32 have been associated with protective immunity against smallpox [8]. In addition to neutralization, antibodies can clear virus-infected cells through antibody-dependent cell-mediated cytotoxicity (ADCC) via interactions with Fc receptors on NK cells [9]. Antibody-mediated ADCC also plays a critical role in antiviral immunity against influenza, SARS-CoV-2, and other viral diseases [10,11,12]. Due to the high sequence and antigenic similarity between vaccinia and MPXV, vaccinia vaccines have also demonstrated protection against MPXV in several preclinical animal studies [13,14,15]. Understanding the antibody response in vaccinated and MPXV-infected cohorts may provide valuable insights for developing effective vaccines against MPXV.

In this study, we used recombinant VTT virus to develop high-throughput luciferase-reporter-based neutralization and ADCC assays to evaluate NAb titers and ADCC responses in healthy and MPXV-infected cohorts.

## 2. Materials and Methods

### 2.1. Cells and Viruses

BHK21, Vero-E6, and 293T cells were maintained in Dulbecco’s Modified Eagle Medium (DMEM, Gibco, Waltham, MA, USA), supplemented with 10% fetal bovine serum (FBS, Gibco), GlutaMAX-1, and penicillin–streptomycin (pen-strep) at a 1:100 dilution (Sartorius, Bohemia, NY, USA). Jurkat cells were maintained in Roswell Park Memorial Institute 1640 (RPMI 1640, Gibco), supplemented with 10% FBS, GlutaMAX-1, and pen-strep. All cells were routinely tested for mycoplasma contamination and were found to be negative. The vaccinia virus Tian Tan (VTT) was a generous gift from the China Centers for Disease Control and Prevention (CDC). VTT virus was propagated on BHK21 cells as previously described [16].

### 2.2. Construction of Recombinant VTT Virus Carrying Reporter Genes

A recombinant VTT virus carrying mCherry and firefly luciferase reporter genes was constructed using CRISPR/Cas9-assisted poxvirus recombination. Briefly, guide RNAs (gRNAs) targeting the TJ2R loci of VTT poxvirus and monkeypox virus were designed using CRISPick (https://portals.broadinstitute.org/gppx/crispick/public (accessed on 2 January 2023)), the gRNA (CACCGTGTGAGCGTATGGC) was selected due to its second-highest “on-target” score and its conservation across both VTT and Mpox viruses, and paired oligonucleotides for the gRNAs were synthesized by GenScript. The gRNA oligonucleotides were annealed and cloned into the LentiCRISPR v2 vector (Addgene #, Watertown, MA, USA) as previously described. 293T cells were transfected with the gRNA vector, and stable transfected 293T cells carrying the gRNA were selected using puromycin. A shuttle plasmid carrying homology arms to the VTT TJ2R loci (GenBank Accession AF095689.1, 5′HR: 79799–80589, 3′HR: 80590–81540) was synthesized by GenScript using the PUC19 vector as a backbone. The mCherry and luciferase reporter genes were subcloned into the shuttle plasmid, under the regulation of the poxvirus EL promoter and the p7.5 promoter, respectively. To generate recombinant VTT virus, 293T cells carrying the poxvirus gRNA were transfected with the shuttle plasmid using EZ Trans (Shanghai Life-iLab Biotech, Shanghai, China) and subsequently infected with VTT virus. After two days of infection, VTT virus from the supernatant and cell lysates was collected. Recombinant VTT viruses expressing mCherry were then purified by cloning, as previously described. The recombinant VTT virus was expanded on BHK-21 cells. The supernatant and lysates from infected cells were collected as stocks of extracellular virions (EVs) and mature virions (MVs), respectively. Virus stocks were aliquoted and stored at −80 °C.

### 2.3. Virus Titration

The recombinant VTT virus stocks were titrated on Vero-E6 cells using the plaque formation method as previously described. Briefly, Vero-E6 cells were seeded in 6-well plates and incubated overnight, followed by incubation with 10-fold serially diluted extracellular virion (EV) or mature virion (MV) virus stocks. After a two-hour infection, the cells were overlayed with 2% agarose and incubated for an additional 36 h. The cells were then fixed and stained with 0.4% crystal violet solution. Plaques were counted, and plaque-forming units (PFUs) were calculated.

### 2.4. Neutralization Assays

The luciferase-based neutralization assay was performed on BHK-21 cells using recombinant VTT virus. Cells were seeded in 96-well plates at a concentration of 10^4^ cells per well and incubated overnight to form a monolayer. Heat-inactivated sera were serially diluted in DMEM supplemented with 2% FBS and 10% guinea pig serum complement (Shanghai Yuanye Bio-Technology, Shanghai, China). Mouse anti-VTT sera, prepared by immunizing C57/B6J mice with paraformaldehyde-inactivated VTT virus, were used as positive controls in the neutralization assays. The diluted sera were mixed with 1000 PFU recombinant VTT MV or EV virus stocks and incubated at 37 °C for 2 h. The sera–virus mixture was then transferred to the BHK-21 cell monolayer and incubated for an additional 12 h. Luciferase expression in infected cells was detected using the Luciferase Assay System (Promega, Madison, WI, USA, E1500) following the manufacturer’s instructions. The neutralization rate was calculated as the percentage of luminescence reduction in wells with serum samples compared to virus controls. NT50 titers were defined as the serum dilution corresponding to a 50% reduction in relative PFUs compared with virus control wells (virus + cells), after subtracting the background from the control group (cells only). NT50 values were calculated using nonlinear regression in GraphPad Prism 10.1.2. The threshold for NAb positivity, based on previous studies, was >20.

For the plaque-based neutralization assay, Vero-E6 cells were seeded in 12-well plates at a concentration of 10^6^ cells per well and incubated overnight to form a monolayer. Heat-inactivated sera were serially diluted in DMEM supplemented with 2% FBS and 10% guinea pig serum complement. The diluted sera were mixed with 150 PFU recombinant VTT MV or EV virus stocks and incubated at 37 °C for 2 h. The sera–virus mixture was then transferred to the Vero-E6 cell monolayer and incubated for an additional 48 h. The cells were fixed and stained with 0.4% crystal violet solution, and plaques were counted. The neutralization rate was calculated as the percentage of plaque reduction in wells with serum samples compared to virus controls. Neutralizing antibody titer (NT50) was defined as the serum dilution corresponding to a 50% neutralization inhibition rate.

### 2.5. ADCC Assay

The magnitude of antibody-induced ADCC was assessed using Jurkat cells expressing FcγRIII (CD16) and an NFAT-inducible reporter gene, measured by the activation of the NFAT pathway in Jurkat cells [17]. Jurkat cells were sequentially transfected with FcγRIII (CD16) and an NFAT-inducible Nano-luc reporter gene via lentiviral transfection, as previously described [18]. Stably transfected cells were selected using blasticidin. The expression of FcγRIII on transfected cells was confirmed by fluorescence activated cell sorting (FACS) analysis. Vero-E6 cells infected with recombinant VTT virus served as target cells. These cells were seeded in 96-well plates at a concentration of 10^4^ cells per well and infected with recombinant VTT virus at a multiplicity of infection (MOI) of 1 for 8 h. The infected cells were then incubated with diluted serum samples at 37 °C for 30 min before the addition of 10^4^ Jurkat effector cells. After 24 h of incubation, the expression of Nano-luc in Jurkat cells was detected using the Nano-Glo^®^ Live Cell Assay (Promega, N2013) following the manufacturer’s instructions. The magnitude of the ADCC response was defined as the fold change in luminescence in wells with serum samples compared to wells without serum or antibodies.

### 2.6. Statistical Analysis

Statistical analyses were performed as previously described in this section. The neutralizing titers of NT50 were calculated using a six-parameter dose–response curve in GraphPad Prism 10.1.2. For descriptive analysis, data were presented as median (interquartile range, IQR) for continuous parameters and frequency (percentage) for categorical variables. Chi-squared and Fisher’s exact tests were used to compare categorical variables, while for continuous variables, the *t*-test was used for normal data and the Mann–Whitney U test for non-normal data. Two-tailed test was used to compare the NT50-to-NanoLUC ratio between the paired samples of different types. *p* values less than 0.05 were considered statistically significant.

## 3. Results

### 3.1. Participant Information

A total of 43 patients diagnosed with laboratory-confirmed MPXV infection, based on positive reverse transcriptase–polymerase chain reaction (RT-PCR) results from blister fluid swabs, were enrolled in this study. The swabs were tested by the Shanghai Center for Disease Control and Prevention (SCDC) between July and August 2023 in Shanghai. Clinical information, including age, gender, and HIV status, is summarized in Table 1. Plasma samples were collected from the patients at the time of discharge (after the disappearance of the rash). The study was conducted in accordance with a clinical protocol (KY-2024-34) approved by the investigational review board (IRB) of SCDC. All participants provided written informed consent, which was approved by the IRB; participants did not receive financial compensation. This study adhered to the Strengthening the Reporting of Observational Studies in Epidemiology (STROBE) guidelines for cohort studies.

Leftover plasma samples from two MPXV-negative cohorts in our previous study were also included as controls. The first cohort comprised 142 healthy individuals enrolled between 2020 and 2021 to study the antibody response to SARS-CoV-2 Omicron variants in vaccinated individuals [19]. The second cohort included 386 serum samples from men who have sex with men (MSM), collected by SCDC between 2020 and 2022 during an HIV prevalence survey. Clinical data for these two cohorts, including age, gender, and HIV status, are also summarized in Table 1.

### 3.2. Construction of Recombinant VTT Carrying mCherry and Luciferase Reporters

**Figure 1:**
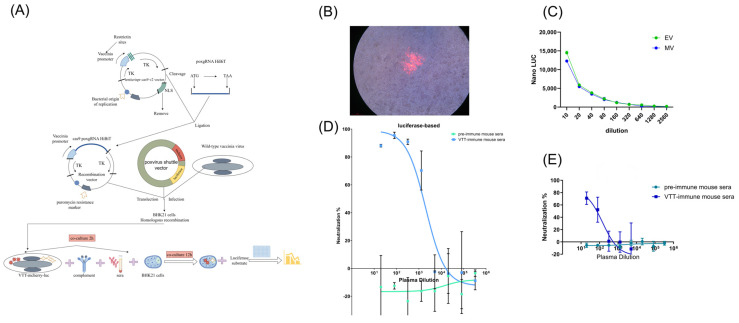
Development of poxvirus-specific neutralization assay with recombinant VTT virus. (**A**) Schematic diagram of construction of recombinant VTT virus expressing mCherry and luciferase dual reporters and development of neutralization assay is shown. (**B**) mCherry expression is observed in BHK21 cells that were infected by recombinant VTT-Luc virus clone. (**C**) Recombinant VTT MV and EV stocks are titrated on BHK21 cells by luciferase expression. (**D**) Neutralization against recombinant VTT virus by mice anti-VTT sera is evaluated by reduction in luciferase expression. Pre-immunized mice sera were used as negative controls. (**E**) Neutralization against recombinant VTT virus by mice anti-VTT sera is evaluated by traditional plaque reduction assay.

To develop a high-throughput neutralization assay, we constructed a recombinant Vaccinia Tian Tan (VTT) virus harboring both mCherry and firefly luciferase reporter genes. A schematic of the construction strategy for the recombinant VTT virus is shown in Figure 1A. To increase recombination efficiency, we employed a CRISPR/Cas9-based approach. Several gRNAs targeting the TJ2R loci of both VTT and monkeypox viruses were designed using CRISPick. The gRNA was synthesized and cloned into the LentiCRISPR v2 vector. 293T cells were transfected with the CRISPR/Cas9 lentivirus and selected using puromycin. Cas9-transfected 293T cells were subsequently infected with VTT virus and transfected with the shuttle plasmid. The VTT-mCherry- luciferase (LUC) clone with the highest luciferase expression was selected and expanded on BHK-21 cells for use in neutralization assays. This recombinant virus maintained high levels of both mCherry and luciferase expression after at least three rounds of expansion on BHK-21 cells (Figure 1B,C), indicating stable incorporation of the reporter genes into the VTT genome. The recombinant VTT virus stock was titrated on BHK-21 cells by luciferase activity and on Vero-E6 cells by plaque formation. As shown in Figure 1C, luciferase expression in infected cells correlated with the PFUs of the input virus stock. Based on luciferase activity, we used 1000 PFUs per well in subsequent neutralization assays.

### 3.3. Validation of Luciferase-Based Neutralization Assay

Before evaluating human plasma and serum samples, we validated the feasibility and robustness of the recombinant VTT virus in the neutralization assay using mouse anti-VTT sera. These sera were collected from mice immunized twice with paraformaldehyde-inactivated VTT viral particles, with pre-immunization sera serving as negative controls. As shown in Figure 1D, sera from all four VTT-vaccinated mice demonstrated potent neutralization of the recombinant VTT virus, with ID50 values ranging from 943.3 to 4295. In contrast, pre-immunization sera showed no neutralization activity against the recombinant VTT virus.

To assess the specificity and sensitivity of the luciferase-based neutralization assay, we conducted a head-to-head comparison with the traditional plaque reduction assay using the same batches of mouse anti-VTT sera and recombinant VTT virus. As shown in Figure 1E, the anti-VTT sera (but not pre-immunized sera) neutralized recombinant VTT viruses in the plaque reduction assay. The ID50 values in the plaque reduction assay ranged from 8.898 to 1695, approximately 10-fold lower than those obtained in the luciferase-based neutralization assay. These results suggest that the luciferase-based assay is consistent with, but more sensitive than, the traditional plaque reduction assay.

### 3.4. Pre-Existing Poxvirus-Specific Nabs Were Detected in MPXV-Negative Cohorts

**Figure 2:**
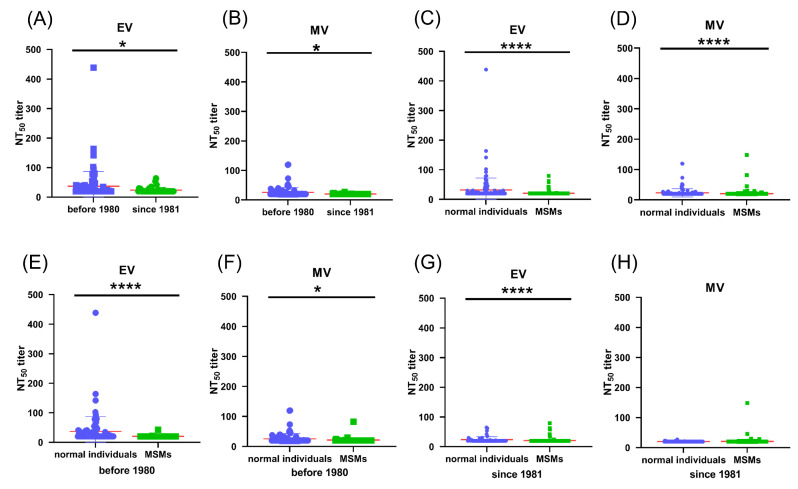
Pre-existing poxvirus-specific Nabs were detected in MPXV-negative individuals. Neutralization titers (NT50) against VTT EV stocks (**A**) or MV stocks (**B**) are shown. Sera from individuals who were born in or before 1980 (*n* = 88) exhibit significantly higher titers of Nabs than those from individuals who were born after 1980 (*n* = 54). NT50 against VTT EV stocks (**C**) or MV stocks (**D**) of sera from MSM group (*n* = 386) are compared with non-MSM group (*n* = 142). NT50 titers against VTT EV stocks (**E**) or MV stocks (**F**) of sera from vaccinia-vaccinated MSM who were born in or before 1980 (*n* = 88) are compared with vaccinia-vaccinated non-MSM group (*n* = 55). NT50 titers against VTT EV stocks (**G**) or MV (**H**) of sera from non-vaccinated MSM who were born after 1980 (*n* = 331) are compared with non-MSM group (*n* = 54). P values were calculated by nonparametric Mann–Whitney U test. * *p* < 0.05, and **** *p* < 0.0001.

We evaluated the poxvirus-specific neutralizing antibody (NAb) response in MPXV-uninfected cohorts. NAb responses in sera from 142 healthy individuals were assessed using the luciferase-based neutralization assay. The NT50 values of each sample against recombinant VTT EV and MV stocks are shown in Figure 2A and Figure 2B, respectively. Since national vaccinia vaccination was halted in 1980 after the eradication of smallpox in China, we divided the cohort into vaccinated and unvaccinated groups based on the donors’ birth years. Of the 142 healthy donors, 88 were born in or before 1980, and 54 were born after 1980. Compared to the unvaccinated group, sera from vaccinated individuals exhibited significantly higher NAb titers against both EV and MV recombinant VTT stocks (Figure 2A,B). Among the vaccinated group, 40% (35/88) of sera samples showed detectable neutralizing antibodies against recombinant VTT stocks, with mean NT50 of 36.77 and 25.52 against EVs and MVs, respectively. In contrast, most sera samples from unvaccinated individuals did not show detectable NAbs against either EV or MV stocks (mean NT50, 23.69, 20.28). These findings indicate that a long-lasting humoral response was provoked in individuals who received vaccinia vaccination over 40 years ago, consistent with previous reports.

### 3.5. Pre-Existing Poxvirus-Specific Nabs in MPXV-Negative MSM Individuals Are Lower than Those in Non-MSM Individuals

The majority of MPXV cases during the 2021–2022 MPXV epidemic were reported in men who have sex with men (MSM) [20,21,22,23]. To understand why MSM may be at higher risk of MPXV infection, we evaluated the neutralizing antibody (NAb) response in MSM and compared it with that of another MPXV-negative cohort. A total of 386 serum samples from MSM were evaluated, and NT50 values against recombinant VTT EV and MV stocks are shown in Figure 2C and Figure 2D, respectively. Compared to the other MPXV-negative cohort, NAb titers in the MSM population were significantly lower. Only 2% (9/386) of serum samples from MSM exhibited detectable NAbs against recombinant VTT. The mean NT50 in the MSM population was 20.57 and 20.66 against EVs and MVs, compared to 31.80 and 23.53 against EVs and MVs in the other MPXV-negative cohort.

We further divided the MSM population into smallpox-vaccinated and unvaccinated groups based on their age. Of the 386 MSM donors, 55 were born in or before 1980. Although they received the same vaccinia vaccination as non-MSM, only a few of the serum samples (3/55) from MSM showed detectable NAbs against either EV (Figure 2E) or MV stocks (Figure 2F). The mean NT50 for MSM was 20.44 and 21.45 for EVs and MVs, which was significantly lower than that observed in non-MSM born in or before 1980 (mean NT50, 36.77 and 25.52). In unvaccinated individuals born after 1980, very few serum samples showed detectable NAbs, and there was no significant difference between MSM and non-MSM groups (Figure 2G,H). These results suggest that the anti-poxvirus immunity induced by vaccination may be weakened in the MSM population, which could explain why MSM appear to be more susceptible to MPXV infection.

### 3.6. VTT-Neutralizing Antibodies Were Induced After MPXV Infection

**Figure 3:**
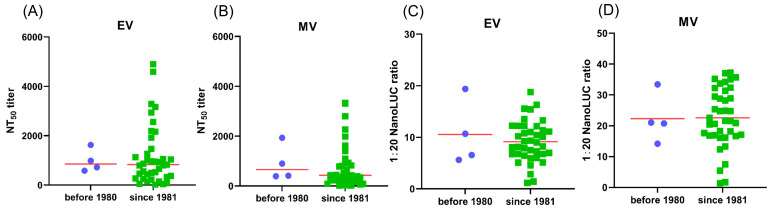
Robust poxvirus-specific antibody responses were provoked after MPXV infection with or without vaccine history. Poxvirus-specific Nabs in sera from MPXV-infected patients who were born in or before 1980 (*n* = 4) are compared with those who were born after 1980 (*n* = 39). NT50 against VTT EV stocks (**A**) or MV stocks (**B**) of sera are shown. ADCC in sera from MPXV-infected patients who were born in or before 1980 (*n* = 4) are compared with those who were born after 1980 (*n* = 39). ADCC response (1:20 NanoLUC ratio) against VTT EV stocks (**C**) or MV stocks (**D**) of sera are shown.

Next, we evaluated the neutralizing antibody (NAb) responses in individuals who had recovered from MPXV infection. A total of 43 patients with laboratory-confirmed MPXV infection were enrolled in the study, and plasma samples were collected at the time of discharge to assess the antibody response. The majority of patients (39/43) were born after 1980 and had not received the vaccinia vaccination. Despite this, these patients developed a robust NAb response following MPXV infection. The antibody titers in plasma from these individuals were comparable to those from vaccinia-vaccinated patients born in or before 1980. There was no significant difference in NAb titers between these two groups, either against EVs (Figure 3A; median NT50 855.00 in vaccinated vs. 833.80 in unvaccinated, mean NT50 979.3 in vaccinated vs. 1177 in unvaccinated, *p* > 0.05) or MVs (Figure 3B; median NT50 657.45 in vaccinated vs. 430.80 in unvaccinated, mean NT50 909.7 in vaccinated vs. 751.4 in unvaccinated, *p* > 0.05).

Antibody-dependent cell-mediated cytotoxicity (ADCC) also plays an important role in inhibiting virus replication. We further evaluated the ADCC response in MPXV-infected patients using a Jurkat cell line stably expressing human FcγRIIIa (CD16a) and the Nano-luciferase reporter gene under the control of NFAT response elements as effector cells. Antibodies capable of inducing ADCC can cross-link FcγRIIIa to activate the downstream NFAT pathway, resulting in an expression of the Nano-luc reporter gene [24], which is detected using the Nano-Glo^®^Live Cell Assay Kit (Promega, E2013). The ADCC response was quantified as the fold increase in luminescence in wells with 1:20 diluted plasma samples, compared to control wells without plasma. As shown in Figure 3C,D, 95.3% (41/43) of plasma samples exhibited ADCC activity when either EV-infected cells (Figure 3C) or MV-infected cells (Figure 3D) were used as targets. Similarly to the NAb response, there was no significant difference in the ADCC response between the vaccinated and unvaccinated groups.

### 3.7. The Impact of HIV Infection on Antibody Response of MPXV-Infected Patients

**Figure 4:**
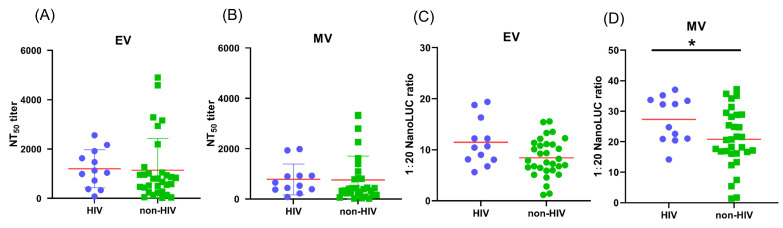
Robust poxvirus-specific antibody response after MPXV infection with or without HIV. Poxvirus-specific Nabs in sera from MPXV-infected patients who are living with HIV (*n* = 12) are compared with non-HIV individuals (*n* = 31). NT50 against VTT EV stocks (**A**) or MV stocks (**B**) of sera are shown. Poxvirus-specific Nabs in sera from MPXV-infected patients who are living with HIV (*n* = 12) are compared with non-HIV individuals (*n* = 31). ADCC response (1:20 NanoLUC ratio) against VTT EV stocks (**C**) or MV stocks (**D**) of sera are shown. P values were calculated by nonparametric Mann–Whitney U test. Two-tailed P values are indicated as * *p* < 0.05.

People living with HIV, particularly those with low CD4 counts (CD4 < 350 cells per mm^3^), accounted for the majority of MPXV-related deaths during the 2022 outbreak [25]. To understand whether antibodies play a role in MPXV-related disease progression, we compared the neutralizing antibody (NAb) and antibody-dependent cell-mediated cytotoxicity (ADCC) responses between MPXV-infected individuals with and without HIV. Among the 43 MPXV-infected patients, 28% (12/43) were HIV-positive. These patients exhibited a robust NAb response (Figure 4A,B). Interestingly, the median NAb titers in HIV-positive patients were slightly higher than in those without HIV infection, both against EVs (median NT50: 1082.50 vs. 881.40, mean NT50: 1200 vs. 1142) and MVs (median NT50: 593.05 vs. 306.75, mean NT50: 780.9 vs. 760.5), although these differences were not statistically significant (*p* = 0.2536 for EVs, *p* = 0.2403 for MVs).

Additionally, higher ADCC responses were observed in HIV-positive patients. As shown in Figure 4C,D, the median fold increase in luminescence in patients with HIV was slightly higher than in those without HIV. The difference was not statistically significant when EV-infected cells were used as targets (median fold change: 10.55 vs. 7.89, *p* = 0.0750), but it was significant when MV-infected cells were used (median fold change: 28.49 vs. 20.60, *p* = 0.0455).

## 4. Discussion

Antibodies play crucial roles in antiviral immunity and are considered key correlates of protection for vaccines against infectious viral diseases, including smallpox, influenza, and SARS-CoV-2 [26,27,28,29,30]. In this study, we developed neutralization and ADCC assays to evaluate the poxvirus-specific antibody responses in individuals both uninfected and infected with monkeypox virus (MPXV). We used recombinant poxviruses carrying luciferase reporter genes for the neutralization assay. The luciferase-based neutralization assay is consistent with the traditional plaque reduction assay but offers superior sensitivity and requires fewer samples. Additionally, it takes just 1–2 days to complete, while the traditional plaque assay typically takes 3–4 days for plaque formation [31]. The luciferase-based assay can be performed in 96-well or 384-well plates, making it suitable for high-throughput applications such as vaccine evaluations for large cohorts or high-throughput screening for neutralizing antibody discovery.

We applied the luciferase-based neutralization assay to evaluate pre-existing poxvirus-neutralizing antibodies (NAbs) in MPXV-negative cohorts, including men who have sex with men (MSM) and non-MSM. NAbs were detected in several individuals from both cohorts, and the presence of these antibodies was associated with the age of the donors. The majority of NAb-positive individuals were born in or before 1980, with significantly higher NAb titers in individuals born in or before 1980 compared to those born after 1980. This observation is consistent with findings from other large cohort studies [6,32]. Given that vaccinia vaccination was a mandatory national program in China until 1980 [33], the presence of poxvirus-specific NAbs in MPXV-negative individuals likely reflects prior vaccinia immunization.

Considering the high antigenic similarity between vaccinia and MPXV [2], pre-existing NAbs induced by vaccinia immunization may provide protection against MPXV infection. In our MPXV-infected cohort, only 4 patients were born in or before 1980 and vaccinated, while the remaining 39 were unvaccinated, underscoring the prophylactic role of pre-existing NAbs in preventing MPXV infection. Additionally, we observed that pre-existing anti-poxvirus immunity appeared to be weakened in the MSM cohort. Compared to the non-MSM cohort, MSM exhibited lower NAb titers and a lower percentage of NAb-positive individuals. Since the majority of MPXV infections during the 2022 outbreak was reported in the MSM population, this diminished immunity may partly explain why MSM are more susceptible to MPXV infection. Further investigation into the factors that weaken anti-poxvirus immunity in MSM populations is warranted.

We also assessed antibody responses in individuals who recovered from MPXV infection. Although the majority of MPXV-infected patients in our cohort were born after 1980 and had not received vaccinia vaccination, most developed robust poxvirus-specific antibody responses after infection. Plasma from these patients not only exhibited neutralization against recombinant VTT virus but also showed ADCC activity against VTT-infected cells. We observed no significant differences in antibody responses between patients born in or before and after 1980. However, as only four patients born in or before 1980 were included in our cohort, and we were unable to determine whether they were MSM, it would be prudent to investigate the impact of prior vaccination history on antibody responses in larger cohorts. Moreover, we found no significant differences in antibody responses between HIV-positive and HIV-negative patients. Notably, all participants in our cohort, including those with HIV, presented with mild symptoms and did not progress to severe disease. It would be valuable to evaluate the antibody responses in MPXV-infected patients with severe disease to determine whether antibodies play a protective role in preventing disease progression.

There are several limitations in this study. First, recombinant VTT virus, rather than the actual MPXV, was used in the neutralization and ADCC assays. While strong NAb and ADCC responses were detected in plasma from MPXV-infected patients, confirming the reliability of these assays, our results should be validated using authentic MPXV to ensure broader applicability. Second, the study includes only 43 MPXV-infected patients with mild symptoms. As noted earlier, further validation in cohorts with a larger and more diverse population, encompassing variations in age, MSM status, HIV infection, and disease progression, is needed. Third, as a retrospective study, we do not have documented vaccination records for these cohorts. The vaccination status of the individuals was determined based on their birth years, which may be different from their actual vaccination status.

## 5. Conclusions

We developed high-throughput neutralization and ADCC assays and used them to evaluate poxvirus-specific antibody responses in MPXV-uninfected and -infected cohorts. Pre-existing neutralizing antibodies were detected in uninfected individuals who had been vaccinated with the vaccinia vaccine but were significantly weakened in the MSM population. Strong NAb and ADCC responses were induced in most MPXV-infected patients with mild symptoms, regardless of prior vaccinia immunization or HIV status. These findings could inform future vaccine development and therapeutic strategies for MPXV.

## Figures and Tables

**Table 1:** The characteristic information of participants in this study.

Variables (*n* [%] or Median [QR])	MPXV-Uninfected Cohort (*N* = 142)	MSM ^1^ Cohort (*N* = 386)	MPXV-Infected Cohort (*N* = 43)
**Sex**			
Female	86 (60.56%)	0 (0%)	0 (0%)
Male	56 (39.44%)	386 (100%)	43 (100%)
**Age (yr)**	47 (20–79)	32 (17–64)	32 (22–59)
**Birth year**			
After 1980	54 (38.03%)	331 (85.75%)	39 (90.70%)
In or Before 1980	88 (61.97%)	55 (14.25%)	4 (9.3%)
**HIV infection**			
HIV	NA ^2^	0 (0%)	12 (27.91%)
Non-HIV	NA ^2^	386 (100%)	31 (72.09%)

^1^ MSM: Men who have sex with men. ^2^ NA: Not available.

## Data Availability

The datasets used and/or analyzed during the current study are available from the corresponding author upon reasonable request.
